# CD276-Positive Circulating Endothelial Cells Do Not Predict Response to Systemic Therapy in Advanced Colorectal Cancer

**DOI:** 10.3390/cells9010124

**Published:** 2020-01-05

**Authors:** Elske C. Gootjes, Jaco Kraan, Tineke E. Buffart, Lotte Bakkerus, Barbara M. Zonderhuis, Cornelis Verhoef, Henk M.W. Verheul, Stefan Sleijfer

**Affiliations:** 1 Department of Medical Oncology VUmc, Amsterdam UMC, Vrije Universiteit Amsterdam, Cancer Center Amsterdam, 1081 HV Amsterdam, The Netherlands; 2 Department of Medical Oncology, Erasmus MC Cancer Institute, 3015 GD Rotterdam, The Netherlands; 3 Department of Gastrointestinal Oncology, Antoni van Leeuwenhoek, 1006 BE Amsterdam, The Netherlands; 4 Department of Surgical Oncology VUmc, Amsterdam UMC, Vrije Universiteit Amsterdam, Cancer Center Amsterdam, 1081 HV Amsterdam, The Netherlands; 5 Department of Surgical Oncology, Erasmus MC–Cancer Institute, 3015 GD Rotterdam, The Netherlands; 6 Department of Medical Oncology, Radboud UMC, 6525 GA Nijmegen, The Netherlands

**Keywords:** circulating endothelial cells, colorectal cancer, CD276^+^, chemotherapy, bevacizumab

## Abstract

CD276 can discriminate between tumor derived and normal CECs (circulating endothelial cells). We evaluated whether CD276^+^CEC is a clinically relevant biomarker to predict response to palliative systemic therapy in patients with metastatic colorectal cancer (mCRC). Samples were prospectively collected from patients with mCRC enrolled in the ORCHESTRA trial (NCT01792934). At baseline and after three cycles of 5-fluorouracil/leucovorin and oxaliplatin ± bevacizumab, CECs were measured by flowcytometry (CD34^+^CD45^neg^CD146^+^DNA^+^; and CD276^+^). A clinically relevant cut-off value of (CD276^+^)CECs was determined as 100% sensitivity (and 80% specificity in 95% confidence interval) identifying patients with progressive disease within 6 months. There were 182 baseline samples and 133 follow up samples available for analysis. CEC and CD276^+^CEC counts significantly increased during treatment from 48 to 90 CEC/4 mL (*p* = 0.00) and from 14 to 33 CD276^+^CEC/4 mL (*p* = 0.00) at baseline and at first evaluation, respectively. CEC and CD276^+^CEC counts were not predictive for poor response (area under the curve (AUC) 0.53 for CEC and AUC 0.52 for CD276^+^CEC). Despite numerical changes during therapy, CEC and CD276^+^CEC counts do not adequately predict poor response to first line palliative systemic therapy in patients with mCRC.

## 1. Introduction

Metastatic colorectal cancer (mCRC) is the third cause of cancer related death worldwide [1]. Modern palliative systemic therapy regimens consisting of 5FU-based combination chemotherapy with oxaliplatin, irinotecan, anti-VEGF targeted therapy, anti-EGFR antibodies, and more recently introduced agents (regorafenib, TAS-102) have improved overall survival up to a median of 30 months [2,3,4,5].

Current first line palliative systemic treatment usually consists of doublet chemotherapy with targeted therapy. A triple chemotherapy regimen like FOLFOXIRI with bevacizumab has demonstrated high response rates in patients with mCRC [6] and may be a valuable option for patients who will not benefit from combination chemotherapy, if these patients could be identified upfront. Currently it is recommended that RAS and BRAF mutated tumors and right sided primary tumors be excluded from anti-EGFR therapy [7,8]. Obviously, there is a clinical need for a biomarker with independent additional value that will predict response to first line systemic therapy. If patients who will not respond to treatment can be predicted with clinically relevant sensitivity and specificity prior to starting systemic therapy, unnecessary treatment can be withheld and toxicity prevented. Furthermore, alternative treatment strategies can be considered for these patients.

Circulating endothelial cells (CECs) are cells detached from damaged vasculature. Compared to healthy controls, CEC values are frequently elevated in patients with disseminated malignancies. Previously, it has been shown that in patients with metastatic cancer, CEC numbers at baseline and changes in CEC numbers during systemic therapy are associated with prognosis. Consequently, enumeration of CEC is considered a promising biomarker in oncology [9]. However, limited data is available on the value of CECs in predicting treatment response.

CECs measured in patients with advanced cancer are thought to derive from both damaged normal vasculature as well as tumor vasculature. Although CECs have been shown to be increased in patients with CRC compared to healthy controls [10,11], this surrogate marker of endothelial damage can increase in a variety of conditions including ischemia, infections, and vascular or autoimmune disease [12,13,14]. Measuring tumor-derived CECs could increase specificity and improve predictive value in cancer. Several tumor endothelial markers have been described in literature based on comprehensive SAGE (serial analysis of gene expression) analysis and mouse models differentiating pathological from physiological angiogenesis [15], with CD276 being a promising candidate as described by Mehran et al. and Kraan et al. [16,17]. Tumor cells express low levels of CD276, but tumor associated endothelial cells express high levels. In mCRC varying results have been published on the relationship of CECs with survival [10,11,13,14,16,18,19]. Direct comparison of the data is difficult because of the use of different CEC identification techniques. 

Our group developed a flowcytometry based detection assay for tumor derived CD276^+^CECs, differentiating CECs from non-endothelial cells (i.e., pericytes) and between normal and malignant vasculature, which enables to distinguish a subpopulation of CECs coming from malignant vasculature in patients with advanced malignancies. CEC counts in healthy donors were median 15 (IQR 17.5) [9,17]. Median CD276^+^CEC counts were 9 cells/4 mL for patients with advanced CRC (range 1–293, *n* = 15) compared to 3 cells/4 mL for healthy donors. The subset of CD276^+^CEC in peripheral blood samples were detectable above the upper limit of normal (ULN) for healthy individuals (>8 cells/4 mL, mean +1.96 SD) in more than 53% of patients with advanced CRC (*n* = 15) [17]. As this subpopulation of CD276^+^CECs and changes therein are likely to reflect better potential effects on tumor vasculature than the total number of CECs, further investigation on the frequency of these cells and their association with outcome in patients with cancer is warranted.

The primary objective of the current study was to establish the prevalence of CD276^+^CECs in patients with mCRC and evaluating the dynamics of CD276^+^CECs during systemic therapy. Furthermore, we evaluated the association of (CD276^+^) CEC counts with clinical parameters. We aimed to determine a clinically relevant cut-off value of the absolute count of CD276^+^CECs at baseline with 100% sensitivity for patients with progressive disease within 6 months of first line palliative systemic therapy, with a specificity of 80% included in the confidence interval. 

## 2. Materials and Methods

Samples were collected as part of the translational study program of the ORCHESTRA trial from May 2013 to July 2018. The ORCHESTRA trial is a randomized multicenter clinical trial for patients with multi-organ, colorectal cancer metastases comparing the combination of chemotherapy and maximal tumor debulking versus chemotherapy alone (NCT01792934).

Written informed consent was obtained from all patients included in the ORCHESTRA trial. Patients were 18 years or older and had an indication for first line palliative systemic therapy for mCRC. Comprehensive in and exclusion criteria are available at clinicaltrials.gov. The trial protocol was approved by the Ethical Committee of the VU university Medical Center in Amsterdam, the Netherlands (no. 2012-073).

All patients received 5-FU/oxaliplatin based systemic therapy ± bevacizumab at physician discretion. Systemic therapy consisted of orally administered capecitabine 1000 mg/m^2^ twice a day for two weeks and oxaliplatin 130 mg/m^2^ intravenous (CAPOX) on day 1 of each 3-week cycle or comparable intravenous regimen consisting of oxaliplatin 85 mg/m^2^ on day 1 and 400 mg/ m^2^ LV followed by 400 mg/m^2^ 5-FU bolus and 2400 mg/m^2^ continuous infusion over 46 h (modified FOLFOX6) of each 2-week cycle. Bevacizumab was added at physician discretion to the CAPOX regimen at a dose of 7.5 mg/kg bevacizumab as intravenous infusion over 30–90 min on day 1. The FOLFOX regimen could be combined with biweekly 5 mg/kg bevacizumab as intravenous infusion over 30–90 min on day 1. After 3 cycles of CAPOX (B) or 4 cycles of FOLFOX (B) a CT scan of thorax and abdomen was performed. Follow up scans were done at least every 3 months. Blood samples were collected at baseline and at first evaluation (after 3 cycles of CAPOX ± B or 4 cycles of FOLFOX ± B). Samples were collected in Cellsave tubes and shipped to central laboratory at Erasmus MC Cancer Institute and processed within 96 h. A multi-color flow cytometry protocol was used to identify endothelial cells [9]. CECs were defined as nucleated cells staining positively with the DNA specific nuclear dye DRAQ5, that express the endothelial markers CD34, CD144, and CD146, and lack the expression of the pan-leukocyte marker CD45. By adding CD276 we identified the subset of tumor derived CECs. Total and CD276^+^CEC were enumerated in a total blood volume of 4 mL as described previously [17].

### 2.1. Clinical Data 

Data were collected on age, gender, location of primary tumor, location, and number of metastases as well as baseline LDH and CEA. Systemic therapy regimen (CAPOX or FOLFOX ± bevacizumab) was documented per patient. The number of organs involved in metastatic disease, number of metastases and baseline LDH were used as indicators of tumor burden. After three cycles of CAPOX (B) or four cycles of FOLFOX (B) response was evaluated according to Response Evaluation Criteria in Solid Tumors (RECIST1.1) and at least every 3 months thereafter. At data cut off, progression free survival at 6 months after study inclusion was documented for all patients.

### 2.2. Statistical Analysis

Median cell counts of (CD276^+^)CECs and interquartile range were reported. Significance levels were calculated with Mann–Whitney U test for dichotomous variables or Kruskal–Wallis for categorical variables. Baseline and follow-up samples were compared by Wilcoxon signed rank test. Areas under the curve were calculated by receiving operating curves. Correlations were calculated by Spearman’s Rho. Univariate logistic regression was used to calculate predictive value for response.

## 3. Results

**Figure 1:**
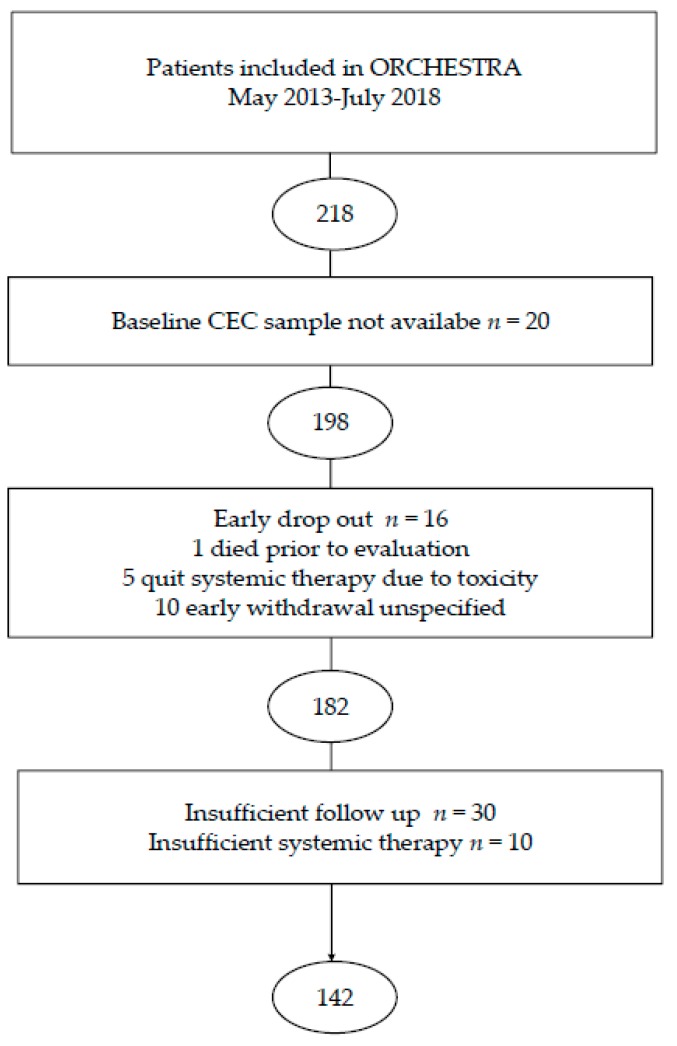
Flowchart of the inclusion of patients for analyses of CECs.

Between May 2013 and July 2018 218 patients were included in the ORCHESTRA trial in the Netherlands. From 20 patients no baseline sample was available and they were excluded from analysis. Sixteen patients did not complete study treatment due to early withdrawal, toxicity or death of unknown cause prior to evaluation (Figure 1). This resulted in 182 patients being evaluable for baseline CEC analysis. From 131 patients, both baseline and follow up samples were available. From 30 patients no sufficient follow up was available to evaluate response to systemic therapy. Eight patients had progressive disease at first evaluation after local treatment as part of the intervention in the ORCHESTRA trial, since chemotherapy was therefore interrupted they were excluded from analysis for response evaluation. Two patients had evident clinical progression prior to the first per protocol evaluation CT scan, both had completed two cycles of systemic therapy and were included in this analysis. An overview of the study inclusion is provided in the flow chart (Figure 1).

### (CD276^+^) CEC Counts

**Figure 2:**
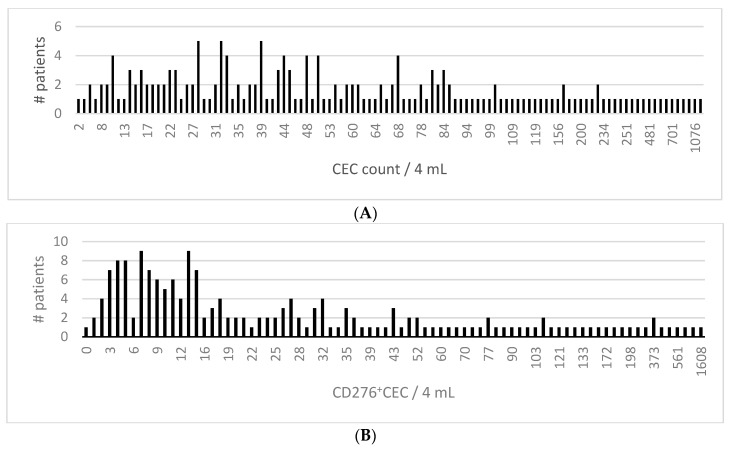
Histograms of (CD276^+^) CEC counts of all available patients (*n* = 142). (**A**) CEC counts per 4 mL; median 48.5 CEC/4 mL (interquartile range 65). (**B**) CD276^+^CEC counts per 4 mL, median 18 CD276^+^CEC/mL (interquartile range 35).

At baseline a median of 48.5 CEC/4 mL (IQR 65) and a median of 18 CD276^+^CEC/mL (IQR 35) was measured (Figure 2). The median subset of CD276^+^CEC from total CECs was 41%. We measured a CD276^+^CEC count higher than the ULN (>8 CECs/4 mL) in 74% of patients. CEC counts for relevant clinicopathological variables revealed no significant differences for both total CEC and CD276^+^CEC counts for any of the variables. Total CEC and CD276^+^CECs counts had no significant correlation with white blood cell count, platelets, LDH, CEA, the number of organs involved in metastatic disease or total number of metastatic lesions (<5, 5–10 or >10). High baseline LDH, involvement of >2 organs or >10 metastases were used as surrogate markers for high tumor burden, but did not show significant differences in (CD276^+^)CEC counts (supplementary Table S1). From a subgroup of 40 patients total volumetric measurements were done. There was poor correlation with total volume and (CD276^+^)CEC counts (CEC spearmans Rho 0.10, *p* 0.15, R^2^ 0.003; CD276^+^CEC Spearman’s Rho 0.17, *p* 0.27, R^2^ 0.006). (data not shown).

**Figure 3:**
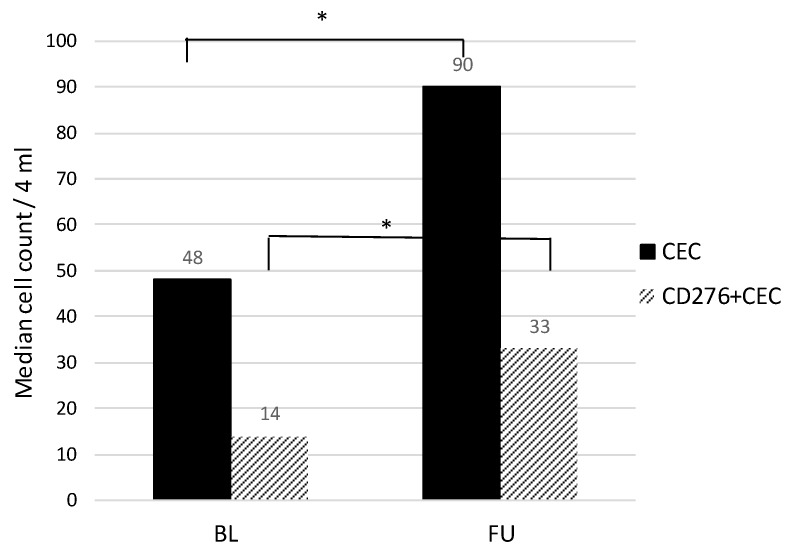
(CD276^+^)CEC dynamics. CEC and CD276^+^CEC counts per 4 mL in baseline and follow up blood samples. * Significant *p* value.

From 131 patients a follow up sample was available. During systemic therapy the CEC count increased significantly (*p* < 0.00) from median 48 to 90/4 mL. The CD276^+^CEC increased significantly (*p* < 0.00) from median 14 to 33/4 mL. The CD276^+^ subset of total CECs decreased from 41% to 36% (*p* = 0.09) (Table 1, Figure 3).

Baseline (CD276^+^)CEC values were not significantly different from patients treated with or without bevacizumab (Supplementary Table S1; CEC *p* = 0.80; CD276^+^CEC *p* = 0.88). In patients treated with bevacizumab, CEC counts were lower after three cycles compared to patients treated with chemotherapy alone 75.5 vs. 131 CEC/4 mL (*p* = 0.04), 28 vs. 41 CD276^+^CEC/4 mL (*p* =0.08). Both in patients treated with doublet chemotherapy alone and patients treated with chemotherapy and bevacizumab a significant increase in total CEC and CD276^+^CEC counts was seen after three cycles of systemic therapy. 

For patients treated with doublet chemotherapy plus bevacizumab (*n* = 98) there was a significant increase in total CEC counts after three cycles of systemic therapy (from median 47 to 75.5/4 mL, *p* = 0.00), and CD276^+^CEC from median 15.5 to 28/4 mL (*p* = 0.01). The CD276^+^ subset of total CECs decreased significantly after treatment from 40% to 35% in the follow up sample (*p* = 0.05). 

For patients treated with only doublet chemotherapy without bevacizumab (*n* = 33) both total CEC as well as CD276^+^CEC counts increased significantly after three cycles of therapy (median CEC from 48 to 131; *p* = 0.03 and median CD276^+^CEC from 14 to 41 *p* = 0.01). The subset was 41 versus 42% respectively (*p* 0.74) (Table 1). 

**Figure 4:**
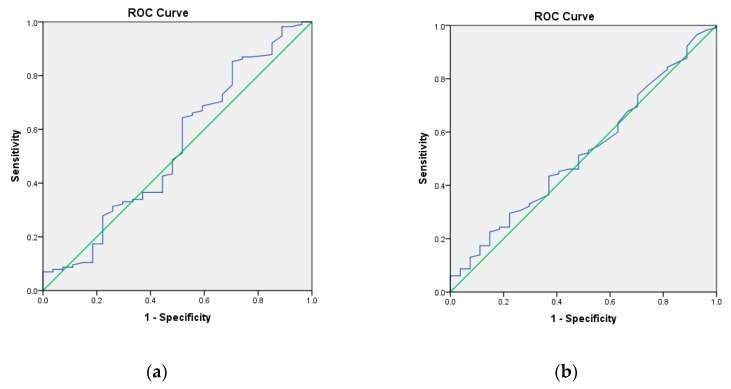
(**a**) Baseline CEC correlation with progression free survival (long versus short); Area under curve (AUC) = 0.533. (**b**) Baseline CD276^+^CEC correlation with progression free survival (long versus short); AUC = 0.524.

None of the circulating endothelial cell measurements, both baseline or follow up CEC and CD276^+^CEC counts, nor the change in CEC count (Delta CD276^+^CEC) could predict poor response to systemic therapy with statistical significance (Table 2). Receiver operating characteristic (ROC) curves for total CEC and CD276^+^CEC in predicting progressive disease within 6 months showed low AUCs of 0.533 for total CEC and 0.524 for CD276^+^CEC (Figure 4). 

## 4. Discussion

Our study was designed to evaluate the prevalence of tumor derived circulating endothelial cells (CD276^+^CEC) measured by flowcytometry in patients with mCRC and explore the predictive value for response to systemic therapy. Previous studies evaluating CEC using (CD34^+^CD45negDNA^+^CD146^+^) for CEC identification focused mainly on predicting survival in patients treated with first line systemic therapy for mCRC [18,19]. Both studies used the CellSearch system (by Veridex^®^, Menarini Silicon Biosystems Inc. Huntington Valley, PA 19006, USA), one showing no prognostic value for PFS or OS [18], the other increase of PFS and OS for baseline CEC counts < 65/mL [19]. In a third prospective series, CEC ≤ 21/4 mL was found to be an independent prognostic factor of poor survival for patients with mCRC amenable for potentially curative surgery, which was of stronger prognostic value than circulating tumor cells [20]. The flow cytometry enumeration we used, as described before by Kraan et al. has the same markers for CEC identification, albeit with different fluorescent antibodies. CEC counts demonstrated to correlate well with CellSearch system counts, with a slightly higher recovery [9]. Other studies used flow cytometry for CEC enumeration with different cluster of differentiation markers. Ronzoni et al. [10], showed increase of PFS and OS for patients with mCRC with CEC count < 40/mL (CD45neg CD146^+^CD34^+^CD133neg) if treated with first line palliative systemic therapy with bevacizumab. Malka et al. [14] showed increase in PFS but not for OS (cut off 23 CEC/mL based on CD31^+^CD146^+^CD45neg7AADneg viable cells/mL by FACS analysis) in 97 patients from the randomized phase II FNCLCC ACCORD 13/0503 trial, receiving first line palliative systemic therapy with bevacizumab with either XELIRI or FOLFIRI. In patients undergoing resection for colorectal liver metastases CEC counts (CD34^+^CD45negCD146^+^) before surgery did not have additional value in predicting 2 year outcome [21]. 

We were able to detect CECs by FACS analysis in all patients with a range from 2–1627 per 4 mL blood and CD276^+^CECs were measured in all but one patient, ranging from 0–1608/4 mL. The median CEC count of 48.5/4 mL (12/mL) is higher than reported by others using the same markers. Simkens et al. reported a median of 6.8 CEC/mL in a large population of 435 patients [18]. Studies from CEC counts prior to liver resections reported 5/mL (CellSearch, 140 patients) or 10 vs. 20/mL for patients with respectively good (*n* = 102) or poor (*n* = 52) outcome, (CD34^+^CD45^−^CD146^+^ measured by FACS). [20,21]. In this study, a significant higher CEC count after three cycles of chemotherapy (*p* = 0.00), with a nearly 2-fold increase (48 to 90/4 mL) was detected. This is in line with the findings of Simkens who reported a 1.5 fold increase (median CEC from 6.8 to 10.5/mL after 6–12 weeks), which was not prognostic for PFS or OS [18].

Our aim was to identify patients who progress within 6 months of start with therapy by (CD276^+^)CEC counts. The AUC of the ROC for CEC counts duration of response < 6 months was 0.53 and 0.52 for CD276^+^CEC counts, indicating that measuring (CD276^+^)CECs is a poor test to predict response to therapy. No clinically relevant cut-off value could be established. Baseline (CD276^+^)CEC counts were not significantly different for any baseline clinicopathological variable. We were not able to validate the cut-off established by Malka et al. [14] who found baseline CEC above the 75th percentile to be an independent prognostic factor for 6 months PFS rate (*p* = 0.44,), nor the finding by matsusaka et al. [19] who found an increase of pFS and OS for patients with a baseline CEC count of <65/mL (*p* 0.347).

There are several potential factors that might confound our results. CD276 could also be detected in normal liver endothelium [16]. As in our cohort 83% of patients had liver involvement in metastatic disease, this could interfere with tumor specificity of the CD276^+^CECs, due to CD276 expression on liver endothelial cells. In contrast with this thought, we did not find a significant difference between CD276^+^CEC counts of patients with or without liver metastases (median 17 vs. 23/4 mL; *p* = 0.33, data not shown). Since patients had to had adequate liver function to be eligible for study participation, important underlying liver disease interfering with CD276^+^CEC counts seems unlikely. Only 5% of patients had > grade 1 elevation of liver enzymes at follow up, possibly reflecting oxaliplatin induced liver toxicity, no important changes in CD276^+^CEC counts were seen in these patients.

Since circulating endothelial cells act as a surrogate marker of endothelial damage, counts can increase in a variety of conditions including ischemia, infections, and vascular or autoimmune disease. All baseline samples were taken prior to start of systemic therapy. Previous adjuvant systemic therapy did not include bevacizumab and was at least 6 months prior to inclusion and is therefore less likely to influence measurements. 

Prior to trial participation, patients with hypertension should have well controlled blood pressure under 160/95 mmHg on a stable antihypertensive regimen (grade II hypertension according to CTCAE 4.03). Patients with uncontrolled infections, a history of congestive heart failure >New York Heart Association class 2 or active coronary artery disease and cardiac arrhythmias requiring anti-arrhythmic therapy were excluded from trial participation (beta blockers or digoxin permitted). Closer evaluation of six patients with baseline (CD276^+^)CEC values > mean +2 SD, revealed no hypertension or a history of peripheral vascular or cerebrovascular disease in these patients. None of these patients had surgery or radiotherapy < 6 weeks prior to baseline measurement.

Patients were not randomized to receive bevacizumab or not. Seventy-six percent of patients were treated with bevacizumab in combination with CAPOX or FOLFOX at physician discretion, which is in line with guidelines adherence in the Netherlands (63–71% use of targeted therapy in first-line treatment) [22]. The reason to withhold bevacizumab was in 42% a (relative) contraindication for anti-VEGF therapy and for 32% due to the fact that bevacizumab administration was no standard practice in the institution. We did not find significant differences in baseline CEC and CD276^+^CEC counts between patients that did or did not receive bevacizumab. 

Kinetics of CECs have been studied with varying results. Murine models showed that VEGF pathway inhibitors can have differential effects on CECs in that inhibition of tumor angiogenesis is associated with an initial increase in mature CECs, followed by a subsequent reduction [16]. We showed a significant increase in CEC and CD276^+^CEC counts during treatment with chemotherapy combined with bevacizumab. The subset of CD276^+^CEC decreased from 40% to 35% (*p* = 0.05). Simkens found an significant increase after 1–2 weeks compared to baseline in patients with mCRC, with no further increase thereafter [18]. Ronzoni et al. [10] did not find a significant change in CEC counts (CD45^−^CD146^+^CD34^+^CD133neg by FACS) for patients with radiologic response. Manzoni et al. [11] showed that patients with an increase in CECs (CD45negCD146^+^D34^+^, CD133neg by FACS) at the sixth cycle of first line chemotherapy in combination with bevacizumab had a better PFS (p 0.009). 

## 5. Conclusions

In conclusion, CD276^+^CEC counts can be measured in patients with mCRC, but no correlation with clinical parameters was demonstrated. Cell counts increased during systemic therapy. In patients treated with bevacizumab, CEC and CD276^+^CEC counts in follow up samples were lower compared to patients not treated with bevacizumab. Despite numerical changes during therapy, (CD276^+^)CEC counts alone do not adequately predict poor response to first line palliative systemic therapy in patients with mCRC. 

## Figures and Tables

**Table 1:** Circulating endothelial cells (CEC) counts/4 mL

	All Paired Samples *n* = 131			Bevacizumab *n* = 98			No Bevacizumab *n* = 33		
	BL ^a^ Median (IQR ^c^)	FU ^b^ Median (IQR ^c^)	*p* Value	BL ^a^ Median (IQR ^c^)	FU ^b^ Median (IQR ^c^)	*p* Value	BL ^a^ Median (IQR ^c^)	FU ^b^ Median (IQR ^c^)	*p* Value
CEC	48 (57)	90 (120)	0.00 *	47 (59)	75.5 (93)	0.00 *	48 (75)	131 (187)	0.03 *
CD276^+^CEC	14 (33)	33 (50)	0.00 *	15.5 (30)	28 (40)	0.01 *	14 (43)	41 (110)	0.01 *
CD276 subset	0.41 (0.33)	0.36 (0.25)	0.09	0.40 (0.30)	0.35 (0.24)	0.05 *	0.41 (0.36)	0.42 (0.27)	0.74

^a^ BL = baseline. ^b^ FU = follow up. ^c^ IQR = Interquartile range. * significant *p* value < 0.05.

**Table 2:** Response prediction of (CD276^+)^CEC.

	PFS ^a^ > 6 Months Median (IQR ^b^)	PFS ^a^ < 6 Months Median (IQR ^b^)	*p*-Value MWU ^c^
*n*	115	27	
Baseline CEC	48 (67)	48 (67)	0.60
Baseline CD276^+^CEC	18 (35)	17 (29)	0.70
Subset	0.43 (0.34)	0.37 (0.36)	0.87
*n*	89	17	
FU ^d^ CEC	76 (131)	131 (98)	0.14
FU ^d^ CD276^+^CEC	25 (51)	42(34)	0.13
FU ^d^ subset	0.36 (0.26)	0.36 (0.28)	0.78
Delta CEC	14 (91.5)	87 (114.5)	0.08
Delta CD276^+^CEC	7 (37)	22 (49)	0.08

^a^ PFS = Progression Free Survival. ^b^ IQR= Interquartile range. ^c^ MWU = Mann-Whitney-U test. ^d^ FU = follow up.

## Supplementary Materials

The following are available online at https://www.mdpi.com/2073-4409/9/1/124/s1, Tabel S1: Baseline Characteristics.

## Appendix A. ORCHESTRA Study Group

**Participating Centers and Principal Investigators of the ORCHESTRA study group.**

Amphia Hospital, Breda, Dept. of Medical Oncology, Albert J Ten Tije
Isala, Zwolle, Dept. of Medical Oncology, Jan Willem B. de Groot
Radboud University Hospital, Nijmegen, Dept. of Medical Oncology, Sandra A. Radema
Meander Medical Center, Amersfoort, Dept. of Medical Oncology, Haiko J. Bloemendal
Noord West ziekenhuisgroep, Alkmaar, Dept. of Medical Oncology, Mathijs P. Hendriks
Hospital Amstelland, Amstelveen, Dept. of Medical Oncology, Annette A. v Zweeden
Fransiscus Gasthuis, Rotterdam, Dept. of Medical Oncology, Paul Hamberg
Haaglanden Medical Center, Den Haag, Dept. of Medical Oncology, Helgi H. Helgason
Bravis Hospital, Roosendaal, Dept. of Medical Oncology, Monique M. Troost
Elisabeth-TweeSteden hospital, Tilburg, Dept. of Medical Oncology, Laurens V. Beerepoot
Antoni van Leeuwenhoek, Amsterdam, Dept. of Medical Oncology, Cecile M.I. Grootscholten
Deventer hospital, Deventer, Dept. of Surgical Oncology, Hans Torrenga
Maasstad hospital, Rotterdam, Dept. of Medical Oncology, Brigitte C.M. Haberkorn
Jeroen Bosch hospital, Den Bosch, Dept. of Medical Oncology, Hans F.M. Pruijt
IJsselland hospital, Capelle aan de Ijssel, Dept. of Surgical Oncology, Maarten Vermaas
Amsterdam Medical Center, Amsterdam, Dept. of Surgical Oncology, Pieter J. Tanis
Admiraal de Ruyter hospital, Goes, Dept. of Medical Oncology, Henk van Halteren
Máxima Medical Center, Eindhoven, Dept. of Medical Oncology, Ard Vreugdenhil
Maastricht University Medical Center, Maastricht, Dept. of Medical Oncology, Rob L.H. Jansen
Sint Antonius hospital, Nieuwegein, Dept. of Medical Oncology, Maartje Los
Medical Center Leeuwarden, Leeuwarden, Dept. of Medical Oncology, Marco B. Polée
Albert Schweitzer hospital, Dordrecht, Dept. of Medical Oncology, Marija Trajkovic-Vidakovic
Dijklander hospital, Hoorn, Dept. of Medical Oncology, Edwin van Breugel
Gelre hospitals, Apeldoorn, Dept. of Medical Oncology, Hielke Meulenbeld
Onze Lieve Vrouwe Gasthuis, Amsterdam, Dept. of Medical Oncology, Jetske M. Meerum Terwogt

## References

[1] Siegel R.L., Miller K.D., Jemal A. (2018). Cancer statistics, 2018. CA: A Cancer J. Clin..

[2] Venook A.P., Niedzwiecki D., Lenz H.J., Innocenti F., Fruth B., Meyerhardt J.A., Schrag D., Greene C., O’Neil B.H., Atkins J.N. (2017). Effect of First-Line Chemotherapy Combined with Cetuximab or Bevacizumab on Overall Survival in Patients With KRAS Wild-Type Advanced or Metastatic Colorectal Cancer: A Randomized Clinical Trial. JAMA.

[3] Heinemann V., von Weikersthal L.F., Decker T., Kiani A., Vehling-Kaiser U., Al-Batran S.E., Heintges T., Lerchenmuller C., Kahl C., Seipelt G. (2014). FOLFIRI plus cetuximab versus FOLFIRI plus bevacizumab as first-line treatment for patients with metastatic colorectal cancer (FIRE-3): A randomised, open-label, phase 3 trial. Lancet Oncol..

[4] Grothey A., Cutsem E.V., Sobrero A., Siena S., Falcone A., Ychou M., Humblet Y., Bouché O., Mineur L., Barone C. (2013). Regorafenib monotherapy for previously treated metastatic colorectal cancer (CORRECT): An international, multicentre, randomised, placebo-controlled, phase 3 trial. Lancet.

[5] Longo-Munoz F., Argiles G., Tabernero J., Cervantes A., Gravalos C., Pericay C., Gil-Calle S., Mizuguchi H., Carrato-Mena A., Limon M.L. (2017). Efficacy of trifluridine and tipiracil (TAS-102) versus placebo, with supportive care, in a randomized, controlled trial of patients with metastatic colorectal cancer from Spain: Results of a subgroup analysis of the phase 3 RECOURSE trial. Clin. Transl. Oncol. Off. Publ. Fed. Span. Oncol. Soc. Natl. Cancer Inst. Mexico.

[6] Cremolini C., Loupakis F., Antoniotti C., Lupi C., Sensi E., Lonardi S., Mezi S., Tomasello G., Ronzoni M., Zaniboni A. (2015). FOLFOXIRI plus bevacizumab versus FOLFIRI plus bevacizumab as first-line treatment of patients with metastatic colorectal cancer: Updated overall survival and molecular subgroup analyses of the open-label, phase 3 TRIBE study. Lancet Oncol..

[7] Stintzing S., Tejpar S., Gibbs P., Thiebach L., Lenz H.J. (2017). Understanding the role of primary tumour localisation in colorectal cancer treatment and outcomes. Eur. J. Cancer.

[8] Douillard J.Y., Siena S., Cassidy J., Tabernero J., Burkes R., Barugel M., Humblet Y., Bodoky G., Cunningham D., Jassem J. (2010). Randomized, phase III trial of panitumumab with infusional fluorouracil, leucovorin, and oxaliplatin (FOLFOX4) versus FOLFOX4 alone as first-line treatment in patients with previously untreated metastatic colorectal cancer: The PRIME study. J. Clin. Oncol..

[9] Kraan J., Strijbos M.H., Sieuwerts A.M., Foekens J.A., den Bakker M.A., Verhoef C., Sleijfer S., Gratama J.W. (2012). A new approach for rapid and reliable enumeration of circulating endothelial cells in patients. J. Thromb. Haemost..

[10] Ronzoni M., Manzoni M., Mariucci S., Loupakis F., Brugnatelli S., Bencardino K., Rovati B., Tinelli C., Falcone A., Villa E. (2010). Circulating endothelial cells and endothelial progenitors as predictive markers of clinical response to bevacizumab-based first-line treatment in advanced colorectal cancer patients. Ann. Oncol. Off. J. Eur. Soc. Med. Oncol..

[11] Manzoni M., Mariucci S., Delfanti S., Rovati B., Ronzoni M., Loupakis F., Brugnatelli S., Tinelli C., Villa E., Falcone A. (2012). Circulating endothelial cells and their apoptotic fraction are mutually independent predictive biomarkers in Bevacizumab-based treatment for advanced colorectal cancer. J. Cancer Res. Clin. Oncol..

[12] Chopra H., Hung M.K., Kwong D.L., Zhang C.F., Pow E.H.N. (2018). Insights into Endothelial Progenitor Cells: Origin, Classification, Potentials, and Prospects. Stem Cells Int..

[13] Mancuso P., Bertolini F. (2010). Circulating endothelial cells as biomarkers in clinical oncology. Microvasc. Res..

[14] Malka D., Boige V., Jacques N., Vimond N., Adenis A., Boucher E., Pierga J.Y., Conroy T., Chauffert B., Francois E. (2012). Clinical value of circulating endothelial cell levels in metastatic colorectal cancer patients treated with first-line chemotherapy and bevacizumab. Ann. Oncol. Off. J. Eur. Soc. Med. Oncol..

[15] Seaman S., Stevens J., Yang M.Y., Logsdon D., Graff-Cherry C., St Croix B. (2007). Genes that distinguish physiological and pathological angiogenesis. Cancer Cell.

[16] Mehran R., Nilsson M., Khajavi M., Du Z., Cascone T., Wu H.K., Cortes A., Xu L., Zurita A., Schier R. (2014). Tumor endothelial markers define novel subsets of cancer-specific circulating endothelial cells associated with antitumor efficacy. Cancer Res..

[17] Kraan J., van den Broek P., Verhoef C., Grunhagen D.J., Taal W., Gratama J.W., Sleijfer S. (2014). Endothelial CD276 (B7-H3) expression is increased in human malignancies and distinguishes between normal and tumour-derived circulating endothelial cells. Br. J. Cancer.

[18] Simkens L.H., Tol J., Terstappen L.W., Teerenstra S., Punt C.J., Nagtegaal I.D. (2010). The predictive and prognostic value of circulating endothelial cells in advanced colorectal cancer patients receiving first-line chemotherapy and bevacizumab. Ann. Oncol. Off. J. Eur. Soc. Med. Oncol..

[19] Matsusaka S., Suenaga M., Mishima Y., Takagi K., Terui Y., Mizunuma N., Hatake K. (2011). Circulating endothelial cells predict for response to bevacizumab-based chemotherapy in metastatic colorectal cancer. Cancer Chemother. Pharmacol..

[20] Rahbari N.N., Scholch S., Bork U., Kahlert C., Schneider M., Rahbari M., Buchler M.W., Weitz J., Reissfelder C. (2017). Prognostic value of circulating endothelial cells in metastatic colorectal cancer. Oncotarget.

[21] Ramcharan K.S., Lip G.Y., Stonelake P.S., Blann A.D. (2015). Increased pre-surgical numbers of endothelial progenitor cells and circulating endothelial cells in colorectal cancer fail to predict outcome. Int. J. Colorectal Dis..

[22] Keikes L., van Oijen M.G.H., Lemmens V., Koopman M., Punt C.J.A. (2018). Evaluation of Guideline Adherence in Colorectal Cancer Treatment in The Netherlands: A Survey Among Medical Oncologists by the Dutch Colorectal Cancer Group. Clin. Colorectal Cancer.

