# Supplementary material for: CD276-Positive Circulating Endothelial Cells Do Not Predict Response to Systemic Therapy in Advanced Colorectal Cancer
Source: Cells. 2020 Jan 5;9(1):124. doi: 10.3390/cells9010124 (PMC7016770; doi:10.3390/cells9010124)
Supplement: Supplementary file 1 [file cells-09-00124-s001.pdf]

Supplementary Table

Table S1. Baseline Characteristics of patients

**Parameter**	**N(%)**	**Median CEC (IQR[1])**	**P value (Mann Whitney U)**	**MeanCD276+CEC (median; IQR[1])**	**p value (Man Whitney)**
Gender			0.96		0.84
Male	118 (66)	48 (59)		17 (35)	
Female	61 (34)	50 (70)		19 (48)	
Age			0.97		0.75
<65	97 (54)	52 (66)		20 (45)	
≥65	82 (46)	47.5 (69)		15 (31)	
Chronicity			0.92		0.57
Synchronous	108 (60)	46.5 (78)		17.5 (31)	
Metachronous	72 (40)	51 (51)		17.5 (40)	
Sidedness of primary tumour			0.86		0.71
Left	129 (70)	48 (62)		17 (29)	
Right	52 (30)	53.5 (69)		21 (51)	
Tumour location			0.87		0.55
Colon	101 (56)	53 (75)		20 (49)	
Rectum	80 (44)	47.5 (54)		17.5 (24)	
Primary in situ			0.94		0.71
Yes	66 (36)	49 (66)		21.5 (35)	
No	116 (64)	48.5 (64)		16 (41)	
Number of metastases			0.78		0.75
<5	39 (22)	56 (64)		22 (53)	
5-10	88 (49)	47.5 (67)		18 (40)	
>10 or diffuse	51 (29)	48 (71)		14 (35)	
Number of organs involved			0.58		0.44
2	107 (59)	52 (77)		21 (44)	
>2	75 (41)	48 (54)		14 (24)	
CEA ug/l			0.90		0.67
>5	135 (78)	50 (67)		18 (36)	
<5	38 (22)	44 (55)		17 (37)	
Chemotherapy[2]			0.23		0.44
CAPOX	171 (94)	50 (68)		19 (42)	
FOLFOX	10 (6)	33.5 (28)		12.5 (13)	
Bevacizumab					
Yes	138 (76)	49.5 (68)	0.90	18 (35)	0.82
No	43 (24)	45 (53)		14 (42)	
Prior (neo) adjuvant chemo			0.44		0.23
Yes	34 (19)	44 (52)		13 (26)	
No	147 (81)	50 (68)		18 (41)	
Prior chemoradiation			0.41		0.55
Yes	31	55 (50)		25 (21)	
No	150	46.5 (70)		17.5 (45)	
Response at first evaluation			0.41		0.81

PD[3]	11	35 (192)	0.41	13 (79)	0.81
Non PD[3]	158	48 (66)		17.5 (35)	
LDH			0.50		0.38
Normal	144 (81)	48 (69)		18 (35)	
Abnormal	33 (19)	50 (50)		14 (36)	
Previous local treatment			0.45		0.29
Yes	59 (33)	50 (81)		23 (44)	
No	121 (67)	48 (58)		17 (35)	
ECOG PS[4]			0.55		0.39
0	115 (73)	48 (58)		18 (33)	
1	43 (27)	48 (70)		22 (42)	

[1]IQR = Interquartile range

[2]CAPOX = combination chemotherapy with capecitabine and oxaliplatin. FOLFOX = combination chemotherapy infusional 5-FU/Leucovorin and oxaliplatin.

[3]PD = progressive disease

[4] Eastern Cooperative Oncology Group Performance Scale
